# Ras-Related Nuclear Protein *Ran3B* Gene Is Involved in Hormone Responses in the Embryogenic Callus of *Dimocarpus longan* Lour.

**DOI:** 10.3390/ijms17060873

**Published:** 2016-06-03

**Authors:** Qilin Tian, Yuling Lin, Dongmin Zhang, Ruilian Lai, Zhongxiong Lai

**Affiliations:** Institute of Horticultural Biotechnology, Fujian Agriculture and Forestry University, Fuzhou 350002, China; tianqilin2009@163.com (Q.T.); buliang84@163.com (Y.L.); qiandanqianlan2011@163.com (D.Z.); lairl0618@163.com (R.L.)

**Keywords:** Ran GTPases, *DlRan3B*, SE, gene expression, promoter deletion analysis

## Abstract

Ras-related guanosine triphosphate (GTP)-binding nuclear protein (Ran) GTPases function as molecular switches and regulate diverse cellular events in eukaryotes. Our previous work suggested that *DlRan3B* is active during longan (*Dimocarpus longan* Lour.) somatic embryogenesis (SE) processes. Herein, subcellular localization of DlRan3B was found to be localized in the nucleus and expression profiling of *DlRan3B* was performed during longan SE and after exposure to plant hormones (indoleacetic acid (IAA), gibberellin A3 (GA_3_), salicylic acid (SA), methyl jasmonte (MeJA), and abscisic acid (ABA)). We cloned and sequenced 1569 bp of 5′-flanking sequence of *DlRan3B* (GenBank: JQ279697). Bioinformatic analysis indicated that the promoter contained plant hormone-related regulatory elements. Deletion analysis and responses to hormones identified stimulative and repressive regulatory elements in the *DlRan3B* promoter. The key elements included those responding to auxin, gibberellin, SA, MeJA, and ABA. DlRan3B was located in the nucleus and accumulated in the late stage of longan SE. The expression of *DlRan3B* was significantly induced by IAA, GA_3_, and ABA, but suppressed by SA and MeJA. Promoter transcription was induced by IAA and GA_3_, but suppressed by SA. Thus, *DlRan3B* might participate in auxin, gibberellin, and ABA responses during longan late SE, and *DlRan3B* is involved in phytohormone responsiveness.

## 1. Introduction

Ran GTPases regulate a multiple series of cell activities by functioning as molecular switches in animals [1,2,3]. In plants, Ran GTPases are involved in various responses, including mediating hormone sensitivities [4,5,6]. Furthermore, overexpression of a Ran GTPase homolog in different plants has caused a variety of developmental abnormalities, such as increases in primordial tissue, mitotic index, sensitivity to exogenous auxin and ABA treatments, and decreases in lateral root number [7,8,9,10]. Meanwhile, the mutations in some Ran GTPase homologs can lead to male sterility [9]. These results have led to a conclusion that *Ran* is essential for plant development and also serve as clues to the potential function of *Ran* in hormone signaling transduction in plants.

Longan tree (*Dimocarpus longan* Lour.) is one of the evergreen fruit trees grown in southern China and its fruit have important applications in food industry and health care [11,12,13]. The development of longan seeds is crucial for longan fruit development, including fruit appearance and processes of fruit setting and ripening. Plant Ran might be involved in the cell activities during plant embryos development, because of its animal its counterparts’ tissue-specific expression during embryogenesis and its role in cell division in animal embryos [14,15,16]. Nevertheless, characterization of plant Ran, especially its involvement in plant embryogenesis and hormone transduction, remains poorly reported.

In previous studies, full-length cDNAs and DNAs of *DlRan3* were cloned from longan somatic embryos and thereafter two 5′ flanking sequences (1256 and 714 bp) of *DlRan3A* (GenBank: JQ775539) and *DlRan3B* (GenBank: JQ279697), respectively, were isolated. It was analyzed by bioinformatics that major elements in the promoters were closely related to phytohormones [17]. A previous study also showed *DlRan3A*’s possible participation in auxin signaling transduction in early stages of somatic embryogenesis (SE) in longan and its possible roles in plant hormone, light, and abiotic stress responsiveness. However, to date, little is known about how *DlRan3B* functions in plant hormone responsiveness in plant embryos.

To further reveal the biological role of *DlRan3B*, its protein subcellular location was observed and its expression profile was analyzed during longan SE processes and under treatments of exogenous plant hormone, including indoleacetic acid (IAA), gibberellin A3 (GA_3_), salicylic acid (SA), methyl jasmonte (MeJA), and abscisic acid (ABA). Furthermore, to understand the regulatory role of *DlRan3B*, its 5′ flanking sequence was isolated and characterized to identify an efficient promoter that could initiate the constitutive expression of a foreign gene in transgenic plants. Furthermore, deletion analysis and different transcriptional activities in response to phytohormones showed that the *DlRan3B* promoter has positive and negative regulatory elements. This study provides a multifaceted view of the potential roles of *DlRan3B* during longan somatic embryo formation and in phytohormone signaling pathways.

## 2. Results

### 2.1. Subcellular Localization of DlRan3B

**Figure 1:**
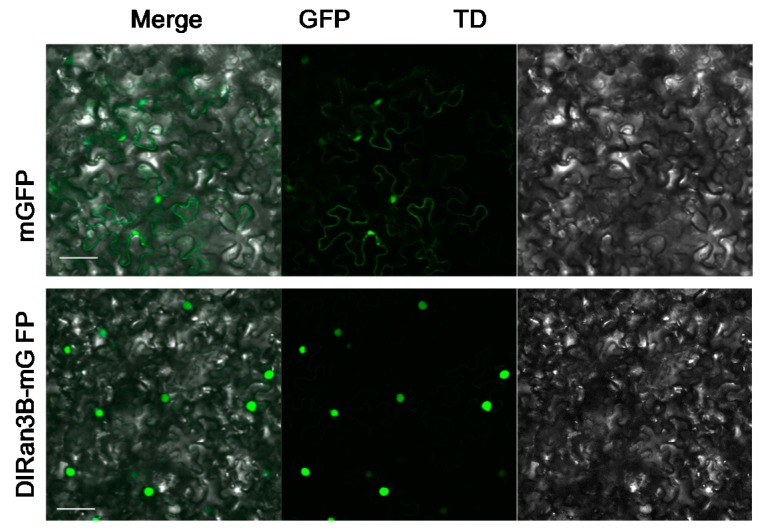
Subcellular localization of DlRan3B. Transient expression of a fluorescent fusion protein (DlRan3B-mGFP (green fluorescent protein)) by agro-infiltration in epidermal cells of tobacco (*Nicotiana benthamiana*) leaves; the upper image (mGFP) is green fluorescence of pCAMBIA1302-GFP and the lower one (DlRan3B-mGFP) is green fluorescence of DlRan3B-mGFP; TD refers to the transmitted light channel. Bars = 50 μm.

A fusion protein of DlRan3B-mGFP (green fluorescent protein) was transiently expressed in the epidermal cells of tobacco leaves to detect subcellular localization of DlRan3B. As a result, DlRan3B-mGFP was dominantly located in the nucleus (Figure 1 and Figure S1).

### 2.2. The Expression Profiling of DlRan3B During Longan Somatic Embryogenesis (SE)

**Figure 2:**
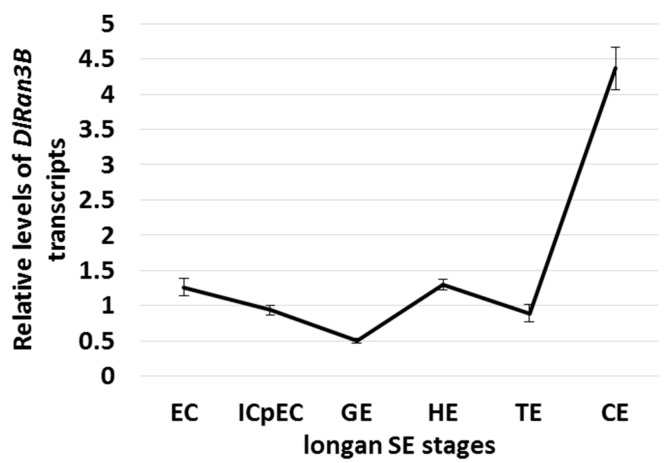
*DlRan3B* expression pattern in longan somatic embryogenesis (SE). Longan SE stages include embryogenic callus (EC), incomplete compact pro-embryogenic cultures (ICpEC), globular embryo (GE), heart-shaped embryo (HE), torpedo-shaped embryo (TE), and cotyledon embryo (CE). The *DlRan3B* expression level was normalized to those of *EF-1a*, *eIF-4a*, and *DLFSD1a*. Data are shown as means ± SD (*n* = 3).

To discover the transcriptional control of *DlRan3B* during longan SE, we measured the transcript levels of *DlRan3B* during longan SE. *DlRan3B* showed decreasing expression during primary developmental stages, with the least *DlRan3B* accumulation observed in the globular embryo (GE) stage, while increasing expression was observed during the middle and late stages of SE, with the highest *DlRan3B* accumulation in the cotyledon embryo (CE) stage. The distinctive pattern suggested an important role of *DlRan3B* accumulation during longan late SE, rather than early stages (Figure 2).

### 2.3. The Effect of Exogenous Plant Hormones on DlRan3B Expression

**Figure 3:**
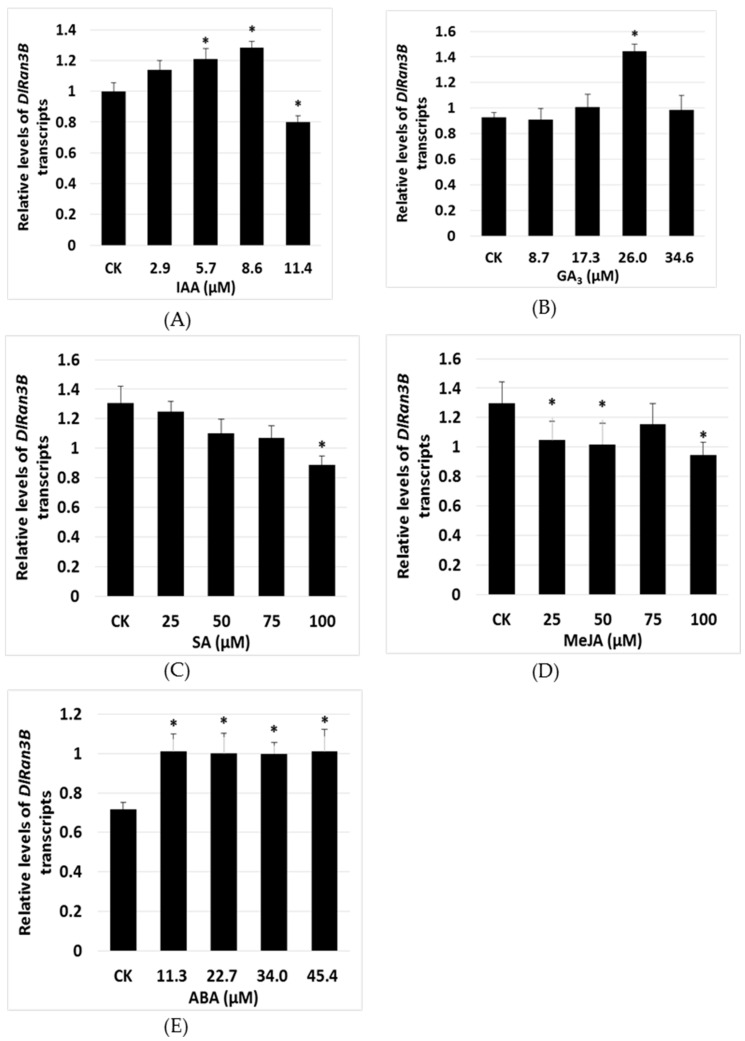
*DlRan3B* expression in response to exogenous plant hormones. (**A**–**E**) ECs were treated with listed hormones at indicated concentrations as follows, (**A**) indoleacetic acid (IAA) (2.9, 5.7, 8.6, and 11.4 μM); (**B**) gibberellin A3 (GA3) (8.7, 17.3, 26.0, and 34.6 μM); (**C**) salicylic acid (SA) (25, 50, 75, and 100 μM); (**D**) methyl jasmonte (MeJA) (25, 50, 75, and 100 μM); or (**E**) abscisic acid (ABA) (11.3, 22.7, 34.0, and 45.4 μM). The DlRan3B expression level was normalized to those of *EF-1a*, *eIF-4a*, and *DLFSD1a*. Data are shown as means ± SD (*n* = 3); the level of significant differences in contrast to the control (CK) are indicated with an asterisk (*) and were assessed by a one-way ANOVA test (* *p* < 0.05).

To understand how *DlRan3B* responds to plant hormones, we analyzed the relative levels of *DlRan3B* mRNA in longan embryogenic calluses (ECs) treated with different concentrations of IAA, GA_3_, SA, MeJA, and ABA (Figure 3A–E). Among the treatments, 26.0 μM GA_3_ enhanced the *DlRan3B* transcript level to approximately 1.6-fold in contrast to the control (Figure 3B); ABA induced a slight, positive transcriptional control on *DlRan3B*, with no dose-dependent effects (Figure 3E). IAA over the range of 2.9–8.6 μM increased *DlRan3B* expression, with 8.6 μM IAA inducing a 1.3-fold level to the control; by contrast, high concentrations of IAA (11.4 μM) inhibited expression (Figure 3A). Notably, *DlRan3B* gene expression showed a sustained decrease as the SA concentration increased (Figure 3C). In addition, a fluctuating expression pattern was found under MeJA treatment (Figure 3D). These results indicated that the transcription of *DlRan3B* responded to plant hormones like auxin, gibberellin, SA, MeJA, and ABA.

### 2.4. The Isolation and Bioinformatic Analysis of the Putative Promoter Region of the DlRan3B Gene

**Figure 4:**
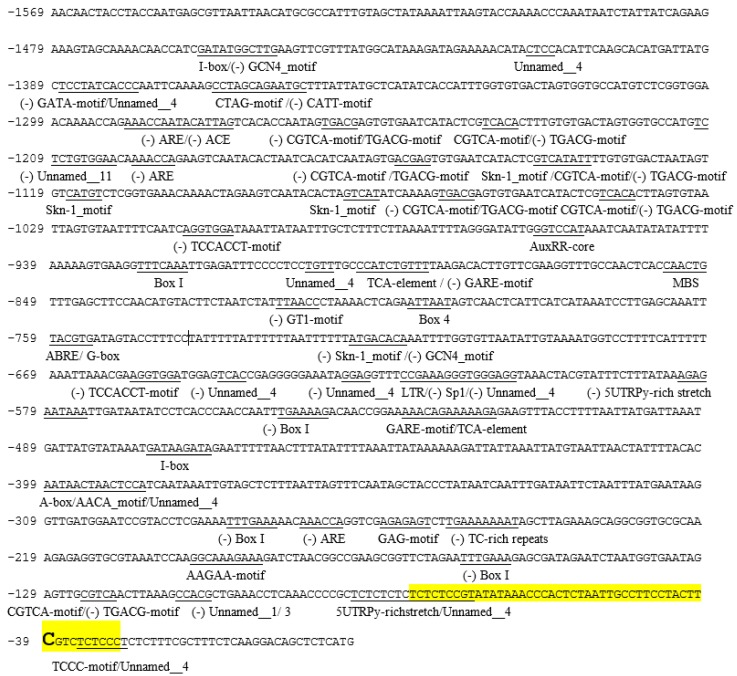
The sequence and structure analysis of the promoter of *DlRan3B.* The translation initiation site is ATG, the core promoter region is highlighted in yellow, and the transcription start sites (TSS) is highlighted in large bold letters.

To figure out the regulatory roles of *DlRan3B*, we further cloned a 1569-bp 5′-flanking sequence upstream of the *DlRan3B* translation initiation site (ATG), covering a previously cloned 5′-flanking sequence of *DlRan3B* (714 bp, GenBank: JQ279697). Thereafter, the Berkeley Drosophila Genome Project and PlantCARE databases were used to predict and analyze potential core sequences and regulatory elements of *DlRan3B* promoter region. Two core sequences in the promoter region were predicted at −952 to −903 (score 0.87) and at −79 to −30 (score 0.97) upstream of the ATG. The potential transcription start sites (TSSs) were T and C, respectively. In the previous study [18], some of the TSSs were proved to be in the region between −79 and −30, but not in the sequence between −952 and −903, thus we proposed that the actual core sequence within the 1569-bp promoter region of *DlRan3B* was located in the region from −79 to −30 and the actual TSS was C (Figure 4). Further analysis identified conserved TATA and CAAT boxes dispersed over the entire promoter sequence. The most frequent motifs observed in the *DlRan3B* promoter were elements involved in light and hormone responses (auxin, gibberellin, SA, MeJA, and ABA). Stress-responsive motifs were also found throughout the promoter region, for example, those involved in low-temperature responsiveness and drought-inducibility (Figure 4, Table 1).

### 2.5. Deletion Analysis of the DlRan3B Promoter

**Figure 5:**
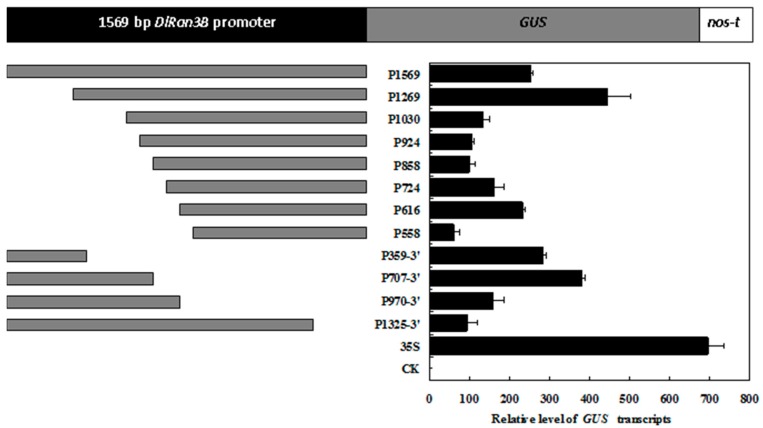
Schematic diagram of the *DlRan3B* promoter deletion constructs. 35S represents the samples with pBI121 vector, and CK represents wild-type tobacco. The *GUS* expression level was normalized to *18SrRNA*. Data are shown as means ± SD (*n* = 3).

To detect the transcription regulation of the *DlRan3B* promoter and figure out key regulatory regions, the constructs of the 1569-bp flanking fragment and a variety of 5′ and 3′ deletions, all fused to *GUS*, a promoterless reporter gene, were prepared (Figure 5). Quantitative real-time PCR (qPCR) assays showed that all of the constructs expressed in tobacco leaves produced relatively lower levels of *GUS* transcripts compared with the control (*CaMV35S*, *35S*) (Figure 5). The difference of *GUS* transcripts between each of the two 5′ deletions showed that activation functional elements were distributed in the positions from −1269 to −924 and −616 to −558, while repression functional elements existed at positions from −1569 to −1269 and −858 to −616. Among the 3′ deletions, positive elements were distributed in the regions from −1211 to −863 and −245 to −1, while inhibitory ones were at −863 to −245. It indicated that both positive and negative functional elements exist in the *DlRan3B* promoter region.

### 2.6. Responsiveness of the DlRan3B Promoter to Hormone Treatments

**Figure 6:**
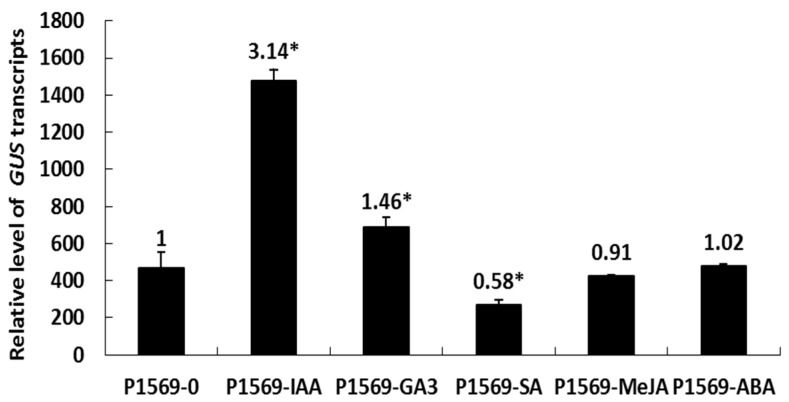
Response of the *DlRan3B* promoter to plant hormones. Tobacco leaves infiltrated with the construct P1569 were under the treatments of 8.6 μM IAA, 34.6 μM GA_3_, 75 μM SA, 100 μM MeJA, 75.7 μM ABA, and water, as a control. Data are shown as means ± SD (*n* = 3). Numbers over the bars represent the fold induction of the five plant hormone treatments over the control; the level of significant differences in contrast to the control (P1569-0) are indicated with an asterisk (*) and were assessed by a one-way ANOVA test (* *p* < 0.05).

The *DlRan3B* promoter contains certain phytohormone-responsive elements, such as ones involved in auxin (AuxRR-core), gibberellin (GARE-motif), SA (TCA-element), MeJA (TGACG- and CGTCA-motif), and ABA (ABRE) responsiveness. To detect the effects of plant hormones on the transcription regulation of *DlRan3B* promoter, 8.6 μM IAA, 34.6 μM GA_3_, 75 μM SA, 100 μM MeJA, and 75.7 μM ABA were used in treating tobacco leaves harboring the construct of *DlRan3B* promoter. The highest *GUS* expression was found in samples under IAA treatment, which showed more than 3-fold higher *GUS* expression compared with the control. GA_3_ also induced *GUS* expression, by about 1.5-fold higher than the control. By contrast, *GUS* expression in the SA-treated samples was repressed. These data demonstrated that regulatory elements related to auxin, gibberellin, and SA responsiveness co-exist in the 1569-bp region of the *DlRan3B* promoter and they are involved in regulating the transcription of a target gene; meanwhile, MeJA and ABA had no significant effect on the transcription regulation of the *DlRan3B* promoter (Figure 6).

## 3. Discussion

### 3.1. DlRan3B Shares Similar Subcellular Localization with Its Longan Homolog and Animal Counterpart

The result of the subcellular localization assay revealed that DlRan3B was exclusively located in the nucleus (Figure 1 and Figure S1), slightly different from DlRan3A, another member in the longan Ran family, which was primarily located in the nucleus, with some in the cytoplasm [6]. The nuclear location of DlRan3B is similar with its longan homolog DlRan3A and animal counterpart. Ran is essential for transporting proteins through the nuclear pore in yeast and mammalian tissue [19,20]. DlRan3B might have a similar nucleocytoplasmic trafficking function to its animal counterparts. However, it remains to be verified what is the relationship between *DlRan3Bs* accumulation and longan late SE, and how DlRan3B functions in embryogenic cells.

### 3.2. DlRan3B Participates in the Auxin, Gibberellin, and ABA Signaling Pathways During Longan Late SE

In the early courses of longan SE, there are high levels of total auxin and gibberellin (IAA + GA_3_), while longan late SE is associated with lower levels of IAA + GA_3_. There was a slight increase in endogenous ABA as SE progressed in longan; during late SE there was a sharp rise in the ratio of ABA/(IAA + GA_3_) to a maximum, rather than a distinct increase in the absolute level of ABA, which suggested the critical role of ABA in the late development of longan somatic embryos [21]. Here, we found that lower doses of IAA and GA_3_ increased the expression of *DlRan3B*, while higher doses did not. Therefore, the low levels of *DlRan3B* transcripts in early longan SE could be attributed to the high levels of auxin and gibberellin. By contrast, *DlRan3B* expression increased to its maximum at the CE stage, probably because of the complex effects of IAA + GA_3_ and ABA. This was different to the expression pattern observed for *DlRan3A* [6], and suggested that *DlRan3B* plays a more important role in longan late SE, involving the hormone signaling pathways of auxin, gibberellin, and ABA. However, during longan SE, the exact nature of this complex relationship among auxin, gibberellin, and ABA has not yet been determined.

### 3.3. The Potential Role of DlRan3B

Ran and Ran-binding proteins have important roles in actively transporting various light and phytohormone receptors, transcription factors, and signaling regulatory proteins into the nucleus when plant cells are exposed to light or phytohormones [4]. However, little is uncovered about the roles of phytohormone in the regulatory network of the *Ran* gene. Similar to *DlRan3A* [6], in the *DlRan3B*’s 5′ flanking sequence, there are many elements involved in light responsiveness (ACE, Box 4, Box I, G-box, GAG-motif, GATA-motif, GT1-motif, I-box, Sp1, and TCCC-motif) and elements involved in hormone responsiveness (ABRE, AuxRR-core, CGTCA-motif, GARE-motif, TCA-element, and TGACG-motif). Here, we found that certain concentrations of IAA and GA_3_ increased *DlRan3B* expression and its promoter transcription activity, which suggested that *DlRan3B* might participate in auxin and gibberellin responsiveness. The auxin-responsive element is located at −1211 to −924 of the *DlRan3B* promoter region and might be a critical regulatory element for stimulating *DlRan3B* expression. A novel role of *Ran* in meristem initiation regulated by auxin signal was observed by overexpressing *TaRAN1* in transgenic *Arabidopsis* and rice, and a close association between Ran and auxin was also discovered in Ran-related proteins [7,22]. Some reports have shown that exogenous GA_3_ upregulates the expression of transcription factors for embryogenesis, and induces the regeneration of somatic embryos, with the acceleration of starch hydrolysis by enhancing α-amylase activity [23,24]. Therefore, *DlRan3B*’s response to GA_3_ and the existence of an A-box (a sequence conserved in alpha-amylase promoters) in the *DlRan3B* promoter suggest the probable involvement of gibberellin in activating nucleocytoplasmic transport of signal elements associated with energy metabolism during longan SE. Nevertheless, little direct evidence is available about the relationship between *Ran* and hormone signaling pathways and about how *Ran* and its promoter function in plant somatic embryos.

The crucial functions of the nuclear trafficking machinery in plant immune and stress signals were reviewed by García and Parker [5]. SA plays a key role in abiotic stress responses, including drought, low temperature, and salinity responsiveness. Some reports have demonstrated that the Ran protein is involved in abiotic stress responses, such as mechanical wounding [25], osmotic stress [9], salinity [26], and cold stress [10,27]. The observation that SA repressed *DlRan3B* expression and its promoter transcription activity correlates with the coexistence of elements involved in drought-inducibility and low-temperature responsiveness in the *DlRan3B* promoter region −858 to −616, which might be negative regulatory elements. Therefore, we hypothesized that SA might participate in the complex interaction of defense-related *cis*-acting elements and trans-acting factors, and the transport of certain transcription factors or mRNA through the nuclear pore via its effect on the *DlRan3B* promoter. In addition, the fact that *DlRan3B* shares ARE (elements involved in anaerobic induction) and TC-rich repeats (elements involved in defense and stress responses) with *DlRan3A* in its promoter region suggested that the longan *Ran* gene family might participate in the response to environmental stress by the mutual effects of different family members. Thus, the ARE, TC-rich repeats, MBS (drought-inducibility), LTR (low-temperature responsiveness), TCA-element (SA responsiveness), and some light-responsive elements, which were located in −1569 to −1269, −858 to −616, and −558 to −245, might be negative regulatory elements. The AuxRR-core (auxin responsiveness), 5′UTR Py-rich stretch (high transcription levels conferring), and some other light-responsive elements, which were located at −1211 to −924, −616 to −558, and −245 to −1, might be positive regulatory elements. The MeJA-responsiveness elements might be involved in complex activation and repression regulation because of their scattered pattern in the promoter region. With environmental stimuli, Ran’s roles in cell activities and nucleocytoplasmic transport of signal elements in plant embryos require further research.

## 4. Materials and Methods

### 4.1. Plant Materials and Nucleic Acid Extraction

Synchronized longan embryogenic cultures from six longan SE stages (*i.e.*, EC, incomplete compact pro-embryogenic cultures (ICpEC), GE, heart-shaped embryo (HE), torpedo-shaped embryo (TE) and CE) were obtained using previously published methods [28,29]. EC were kept on Murashige and Skoog (MS, Phytotechnology M519) medium (2% sucrose, 6 g/L agar, pH 5.8) supplemented with 4.5 μM 2,4-Dichlorophenoxyacetic acid (2,4-D), and MS medium supplemented with 4.5 μM 2,4-D, 2.3 μM kinetin, and 5 mg/L AgNO_3_, subcultured every 20 ds, alternatively. Synchronized GE and the other four embryogenic cultures were obtained by transferring EC to MS medium supplemented with 0.45 μM 2,4-D and MS medium, respectively. All cultures were kept in the dark and at the temperature of 25 °C, with more than three replicates. Tobacco (K326 and *Nicotiana benthamiana*) plants were grown in a growth chamber at 25 °C under a 16/8-h photoperiod (100 μmol/m^2^/s). Genomic DNA and total RNA were extracted from the above-mentioned samples as previously described [6,17].

### 4.2. Subcellular Localization

The subcellular localization assay was conducted as previously described [6]. *DlRan3B* cDNA was fused to the N-terminus of the GFP in the plasmid of pCAMBIA1302. Transient transformation of fluorescent fusion protein by agro-infiltration in epidermal cells of tobacco (*Nicotiana benthamiana*) leaves was performed as previously executed and the subcellular localization of DlRan3B-mGFP was analyzed by laser scanning confocal microscopy (Olympus (Tokyo, Japan); FV1200) [6].

### 4.3. Phytohormone Treatments on Longan EC Samples

After subculturing for 18 days, longan EC (0.2 g) was transferred to 40-mL MS liquid medium (2% sucrose) supplemented with IAA (2.9, 5.7, 8.6, and 11.4 μM), GA_3_ (8.7, 17.3, 26.0, and 34.6 μM ), SA (25, 50, 75, and 100 μM), MeJA (25, 50, 75, and 100 μM), and ABA (11.3, 22.7, 34.0, and 45.4 μM), under agitation at 120 rpm at 25 °C under dark for 24 h, with three replicates. Controls were EC transferred to MS liquid medium. All samples of the above-mentioned treatments were frozen in liquid nitrogen immediately and stored at −80 °C for RNA extraction.

### 4.4. Isolation of DlRan3B Promoter and Bioinformatic Analysis

Genome walking technique was conducted by thermal asymmetric interlaced PCR (Tail-PCR, Takara, Otsu, Japan) to acquire a longer *DlRan3B* 5′-flanking sequence. Each of the three nested PCR amplifications used forward primer AP (AP1, AP2, AP3, or AP4) and specific reverse primers (DlRan3B-pro2-SP1: CGGATTCCATCAACCTTATTCATAAATTAG, DlRan3B-pro2-SP2: GTTGTCT TTTCAAATTGGTTGGGTGAGG, and DlRan3B-pro2-SP3: CGTAGTTTACCTCCCACCCTTTCGG) to obtain the flanking sequence of *DlRan3B*. The cycling conditions of all PCR amplifications were similar to those of Lin’s protocol [30], with some modifications. The potential core promoter sequences and functional elements of the *DlRan3B* promoter were predicted by the Berkeley Drosophila Genome Project and PlantCARE databases [31].

### 4.5. Construction of the PdlRan3B::GUS Fusion Vector and Agrobacterium-Mediated Transient Assay

*Agrobacterium*-mediated transient transformation of epidermal cells in tobacco leaves is a relatively fast technique to determine expression of genes of interest [32]. The 1569 bp of the flanking region, a series of nested 5′ deletions of the *PdlRan3B* fragments (1269, 1030, 924, 858, 724, 616, and 558 bp), and nested 3′ deletions of the *PdlRan3B* fragments (1325, 970, 707, and 359 bp) were amplified from the *DlRan3B* promoter sequence (GenBank accession No. JQ279697) using longan DNA as the template. Longan DNA was extracted from EC using the modified cetyl trimethylammonium bromide method [33]. Forward primers, Del-F1, Del-F2, Del-F3, Del-F4, Del-F5, Del-F6, Del-F7, and Del-F8, were designed to correspond to the 1569, 1269, 1030, 924, 858, 724, 616, and 558 bp sequences of the 5′ deletions (P1569, P1269, P1030, P924, P858, P724, P616, and P558) and the reverse primer Del-R1 was located in the 3′ end of the *DlRan3B* promoter (Table 2). Reverse primers, Del-R2, Del-R3, Del-R4, and Del-R5, plus Del-F1, were designed to correspond to the 1325, 970, 707, and 359-bp sequences of the 3′ deletions (P1325-3′, P970-3′, P707-3′, and P359-3′) (Table 2). *Hin*dIII and *Bam*HI restriction enzyme sites (underlined in Table 2) were introduced at the 5′ end of each forward and reverse primer, respectively. The ligation, *Agrobacterium* transformation, and *Agrobacterium*-mediated transient assay in tobacco were conducted as described previously [6].

### 4.6. Phytohormone Treatments of Tobacco Leaves

To examine the effects of phytohormone on the transcription regulation of the *DlRan3B* promoter, tobacco leaves infiltrated with *Agrobacterium* harboring *DlRan3B* promoter were sprayed with 8.6 μM IAA, 34.6 μM GA_3_, 75 μM SA, 100 μM MeJA, or 75.7 μM ABA for 48 h, with sterile water as a control. All tobacco plants were grown and sampled as above described.

### 4.7. qPCR Analysis

qPCR was conducted to investigate the transcript levels of the *DlRan3B* gene during longan SE, under a series of plant hormone treatments, and transient expression of the *GUS* gene in agro-infiltrated tobacco leaf samples, as described above. Total RNA extraction, determination, and cDNA synthesis were conducted as described previously [6]. qPCR of the *DlRan3B* gene was performed using gene-specific primers (F: CATCATGAAGCTGAGCTTGC; R: CCAGCCTGCAACTGTTCTC), with *EF-1α*, *elF-4α*, and *DlFSD1α* as the reference genes [30,34]. Relative transcript levels of the *GUS* gene were detected using primers (F: CCTGCGTCAATGTAATGTTCTG; R: TTCTCTGCCGTTTCCAAATC), with *18SrRNA* as the reference gene of *18SrRNA* (F: CCTGAGAAACGGCTACCACAT; R: CACCAGACTTGCCCTCCA).

## Figures and Tables

**Table 1:** Prediction of regulatory elements in the 5′-flanking sequence of *DlRan3B*.

Regulatory Element	Function	Sequence
5UTR Py-rich stretch	*cis*-acting element conferring high transcription levels	TTTCTTCTCT/TTTCTCTCTCTCTC
A-box	sequence conserved in alpha-amylase promoters	AATAACAAACTCC
AACA-motif	involved in endosperm-specific negative expression	TAACAAACTCCA
ABRE	*cis*-acting element involved in the abscisic acid responsiveness	TACGTG
ARE	*cis*-acting regulatory element essential for the anaerobic induction	TGGTTT
ACE	*cis*-acting regulatory element involved in light responsiveness	CTAACGTATT
AuxRR-core	*cis*-acting regulatory element involved in auxin responsiveness	GGTCCAT
Box 4	part of a conserved DNA module involved in light responsiveness	ATTAAT
Box I	light responsive element	TTTCAAA
CAAT-box	common *cis*-acting element in promoter and enhancer regions	CAAT/CAAAT
CGTCA-motif	*cis*-acting regulatory element involved in the MeJA-responsiveness	CGTCA
G-box	*cis*-acting regulatory element involved in light responsiveness	CACGTA
GAG-motif	part of a light responsive element	AGAGAGT
GARE-motif	gibberellin-responsive element	AAACAGA
GATA-motif	part of a light responsive element	GATAGGA
GCN4-motif	*cis*-acting element involved in endosperm expression	CAAGCCA/TGTGTCA
GT1-motif	light responsive element	GGTTAA
I-box	part of a light responsive element	GATATGG/GATAAGATA
LTR	*cis*-acting element involved in low-temperature responsiveness	CCGAAA
MBS	v-myb avian myeloblastosis viral oncogene homolog (MYB) binding site involved in drought-inducibility	CAACTG
Skn-1-motif	*cis*-acting regulatory element required for endosperm expression	GTCAT
Sp1	light responsive element	CC(G/A)CCC
TATA-box	core promoter element around −30 of transcription start	TATA/TAATA/TTTTA
TC-rich repeats	*cis*-acting element involved in defense and stress responsiveness	ATTTTCTTCA
TCA-element	*cis*-acting element involved in salicylic acid responsiveness	CCATCTTTTT/CAGAAAAGGA
TCCC-motif	part of a light responsive element	TCTCCCT
TGACG-motif	*cis*-acting regulatory element involved in the MeJA-responsiveness	TGACG

**Table 2:** Sequences of primers used to amplify the *DlRan3B* promoter deletion constructs.

Primer	Sequence (5′ to 3′)	Corresponding Construct	Transgenic Line
Del-F1	TGATTACGCCAAGCTTAACAACTACCTACCAATGAGCG	Full-length (P1569)	P1569
Del-R1	GACCACCCGGGGATCCGAGAGCTGTCCTTGAGAAAGCG	Full-length (P1569)	P1569
Del-F2	TGATTACGCCAAGCTTCCAATAGTGACGAGTGTGAATC	5′Δ (P1269)	P1269
Del-F3	TGATTACGCCAAGCTTATTAGTGTAATTTTCAATCAGGTGG	5′Δ (P1030)	P1030
Del-F4	TGATTACGCCAAGCTTCAAATTGAGATTTCCCCTCCTG	5′Δ (P924)	P924
Del-F5	TGATTACGCCAAGCTTCACCAACTGTTTGAGCTTCCAAC	5′Δ (P858)	P858
Del-F6	TGATTACGCCAAGCTTTTTTTTATGACACAAATTTTGGTG	5′Δ (P724)	P724
Del-F7	TGATTACGCCAAGCTTAGGGTGGGAGGTAAACTACG	5′Δ (P616)	P616
Del-F8	TGATTACGCCAAGCTTCCCAACCAATTTGAAAAGACAACC	5′Δ (P558)	P558
Del-R2	GACCACCCGGGGATCCATTTTTTTCAAGACTCTCTCGACC	3′Δ (P1325-3′)	P1325-3′
Del-R3	GACCACCCGGGGATCCAGTTTACCTCCCACCCTTTCG	3′Δ (P970-3′)	P970-3′
Del-R4	GACCACCCGGGGATCCGGCAAACCTTCGAACAAGTGTC	3′Δ (P707-3′)	P707-3′
Del-R5	GACCACCCGGGGATCCACATGGCACCACTAGTCACAC	3′Δ (P359-3′)	P359-3′

Sequences underlined indicate the restriction enzyme sites of *Hin*dIII and *Bam*HI.

## Supplementary Materials

Supplementary materials can be found at http://www.mdpi.com/1422-0067/17/6/873/s1.

## Abbreviations

Ran	Ras-related nuclear protein
GTP	guanosine triphosphate
NE	nuclear envelope
SE	somatic embryogenesis
IAA	indoleacetic acid
GA_3_	gibberellin A3
SA	salicylic acid
MeJA	methyl jasmonate
ABA	abscisic acid
qPCR	quantitative real-time PCR
EC	EC: embryogenic callus
ICpEC	incomplete compact pro-embryogenic cultures
GE	globular embryos
HE	heart-shaped embryos
TE	torpedo-shaped embryos
CE	cotyledon embryos
GFP	green fluorescent protein
TSS	transcription start site
MYB	v-myb avian myeloblastosis viral oncogene homolog
35S	*CaMV35S*
2,4-D	2,4-Dichlorophenoxyacetic acid
DAPI	4',6-diamidino-2-phenylindole
