# Supplementary material for: Ras-Related Nuclear Protein Ran3B Gene Is Involved in Hormone Responses in the Embryogenic Callus of Dimocarpus longan Lour
Source: Int J Mol Sci. 2016 Jun 3;17(6):873. doi: 10.3390/ijms17060873 (PMC4926407; doi:10.3390/ijms17060873)
Supplement: Supplementary file 1 [file ijms-17-00873-s001.pdf]

Supplementary Materials: Ras-Related Nuclear Protein *Ran3B* Gene Is Involved in Hormone Responses in the Embryogenic Callus of *Dimocarpus longan* Lour.

Qilin Tian, Yuling Lin, Dongmin Zhang, Ruilian Lai and Zhongxiong Lai

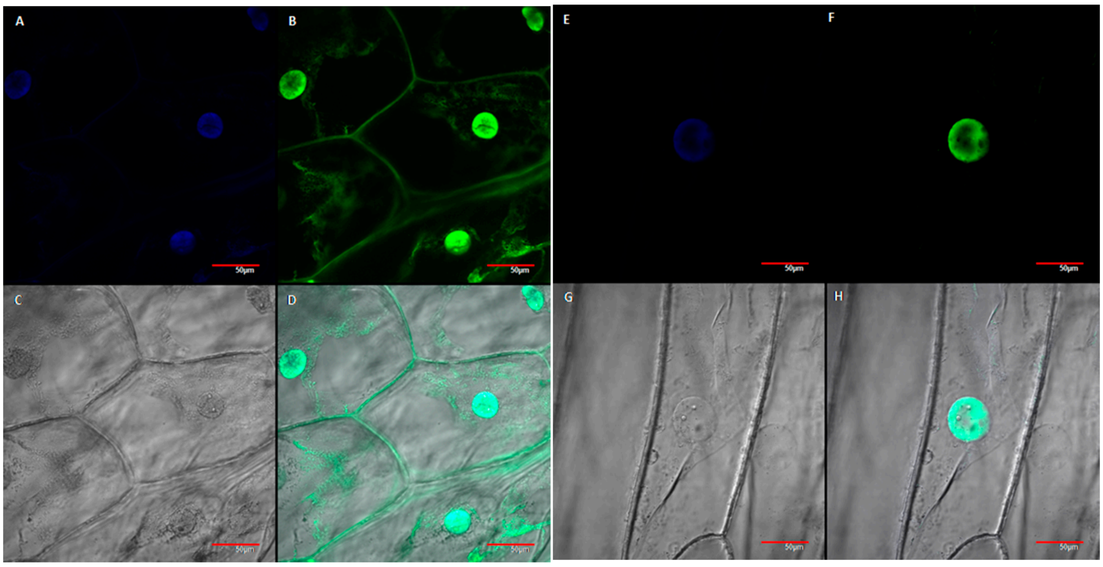

Figure S1. Subcellular localization of DlRan3B (**A**–**H**). Onion epidermal cells were transformed with constructs of pCAMBIA1302-mGFP (green fluorescent protein) (**A**–**D**) and DlRan3B-mGFP (**E**–**H**); DAPI (4′,6-diamidino-2-phenylindole) staining images (**A**,**E**), fluorescence image (**B**,**F**), bright field images (**C**,**G**), and the merged images of fluorescence and DAPI (**D**,**H**); Bars = 50 μm.
